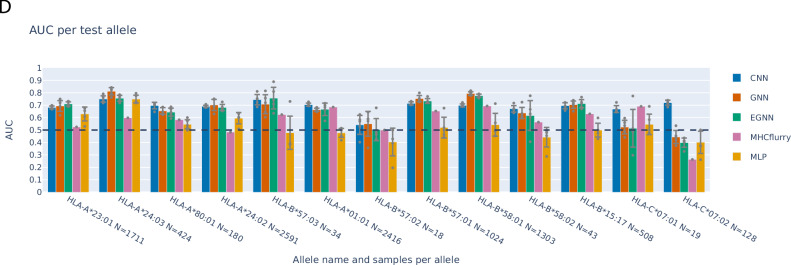# Author Correction: Geometric deep learning improves generalizability of MHC-bound peptide predictions

**DOI:** 10.1038/s42003-025-08422-z

**Published:** 2025-07-05

**Authors:** Dario F. Marzella, Giulia Crocioni, Tadija Radusinović, Daniil Lepikhov, Heleen Severin, Dani L. Bodor, Daniel T. Rademaker, ChiaYu Lin, Sonja Georgievska, Nicolas Renaud, Amy L. Kessler, Pablo Lopez-Tarifa, Sonja I. Buschow, Erik Bekkers, Li C. Xue

**Affiliations:** 1 Medical BioSciences department, Radboudumc, Radboud University Medical Center, 6525 GA Nijmegen, The Netherlands; 2 Netherlands eScience Center, Amsterdam, The Netherlands; 3 University of Amsterdam, Amsterdam, The Netherlands; 4 Department of Gastroenterology and Hepatology, Erasmus MC, University Medical Center Rotterdam, 3015 GD Rotterdam, The Netherlands

**Keywords:** Tumour vaccines, MHC class I, Hepatitis B virus, Molecular modelling

Correction to: *Communications Biology* 10.1038/s42003-024-07292-1, published online 19 December 2024

In the version of the article initially published, due to an inconsistency in the IDs assigned to the binding affinity cases in one post-processing step for the CNN outputs, the previously reported performances of the CNN in Fig. 2D were erroneous. The amended plot represents the correct per-allele performances for the CNN, which are more consistent with the performances of the other networks, especially for HLA-B*57:02 and HLA-C*07:02. The other networks’ performances were correct in the previous version of this plot and are therefore unchanged in this updated version, as shown in Fig. 1. Also, the order of the alleles on the *x* axis has been updated to more correctly represent the distance from the training set, using more decimal values to better separate alleles with similar distance.

Fig. 1 Original Fig. 2D 
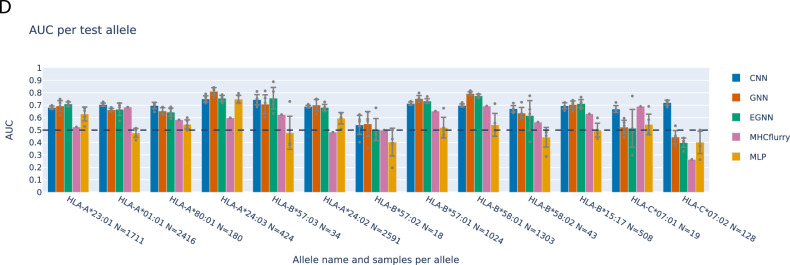


Fig. 1 Corrected Fig. 2D