# Supplementary material for: Geometric deep learning improves generalizability of MHC-bound peptide predictions
Source: Commun Biol. 2024 Dec 19;7:1661. doi: 10.1038/s42003-024-07292-1 (PMC11659464; doi:10.1038/s42003-024-07292-1)
Supplement: Supplementary file 3 — Description of Additional Supplementary Files [file 42003_2024_7292_MOESM3_ESM.pdf]

Description of Additional Supplementary Files

File name: Supplementary Video 1
Description: Video showing the HBV case study peptide HLYSHPIILG in complex with HLA-A*02:01
